# The Role of Verbal Instruction and Visual Guidance in Training Pattern Recognition

**DOI:** 10.3389/fpsyg.2017.01473

**Published:** 2017-09-05

**Authors:** Jamie S. North, Ed Hope, A. Mark Williams

**Affiliations:** ^1^Expert Performance and Skill Acquisition Research Group, School of Sport, Health, and Applied Science, St. Mary’s University Twickenham, United Kingdom; ^2^School of Sport and Exercise Sciences, Faculty of Science, Liverpool John Moores University Liverpool, United Kingdom; ^3^Department of Health, Kinesiology, and Recreation, College of Health, University of Utah, Salt Lake City UT, United States

**Keywords:** familiarity detection, anticipation, perceptual training, expertise

## Abstract

We used a novel approach to examine whether it is possible to improve the perceptual–cognitive skill of pattern recognition using a video-based training intervention. Moreover, we investigated whether any improvements in pattern recognition transfer to an improved ability to make anticipation judgments. Finally, we compared the relative effectiveness of verbal and visual guidance interventions compared to a group that merely viewed the same sequences without any intervention and a control group that only completed pre- and post-tests. We found a significant effect for time of testing. Participants were more sensitive in their ability to perceive patterns and distinguish between novel and familiar sequences at post- compared to pre-test. However, this improvement was not influenced by the nature of the intervention, despite some trends in the data. An analysis of anticipation accuracy showed no change from pre- to post-test following the pattern recognition training intervention, suggesting that the link between pattern perception and anticipation may not be strong. We present a series of recommendations for scientists and practitioners when employing training methods to improve pattern recognition and anticipation.

## Introduction

The ability to think ahead and anticipate future events consistently distinguishes expert performers from their less-expert counterparts (Triolet et al., 2013). In domains such as the military, aviation, and invasion sports, the importance of anticipation is magnified given the dynamic nature and strict time constraints under which performers must make decisions before executing complex motor skills. The ability to utilize perceptual–cognitive processes to inform decision-making and motor actions has been proposed to be a key factor that distinguishes expert performers from those less-expert across domains (Williams et al., 2011).

At a conceptual level, following extended domain-specific practice, experts develop highly specialized and refined knowledge structures which enable them to disregard non-relevant information and attend to only the most critical cues within the display (cf., Ericsson and Kintsch, 1995). These differences are believed to underpin the expert’s ability to identify advance cues in the environment (Savelsbergh et al., 2002), as well as the localized relative motion information between these cues (Diaz et al., 2012), to assess the likelihood of situational probabilities (Farrow and Reid, 2012), and to perceive patterns in displays comprising multiple discrete features (North et al., 2011). Experts encode information more efficiently and effectively, resulting in quicker and more accurate decisions and superior motor execution when compared with novice or less-expert individuals who have accrued less practice.

Perception and knowledge of patterns is typically assessed using recall and recognition paradigms. In the former, participants must recall the positions of display features after a brief exposure, whereas in the latter familiarity judgments are made as to whether or not stimuli have been previously viewed. The typical finding is that experts show a memory advantage for structured stimuli (representing typical formations that one would expect to see), but that this advantage is lost when attempting to recall or recognize unstructured stimuli (where information is randomly organized). These results were originally reported in the domain of chess (De Groot, 1965; Chase and Simon, 1973; Goldin, 1978, 1979), but subsequently the findings have been replicated across multiple domains including diagnostic imaging (Nodine and Kundel, 1987), business (McKelvie and Wiklund, 2004), and in numerous sports such as basketball (Allard et al., 1980), field hockey (Starkes, 1987), Australian Rules Football (Berry et al., 2004), and soccer (Williams and Davids, 1995). It is believed that the ability to quickly recall or recognize previously encountered situations “buys time” and facilitates more accurate anticipation judgments. In dynamic sports like soccer it is likely that participants will never truly encounter the *exact* same situation more than once; however, it is proposed that the critical features of patterns will remain consistent with some room for variability (see Gobet and Simon, 1996). Expert performers are proposed to perceive and encode these key features and relations in displays when recognizing patterns (North et al., 2017). In other words, judging the current situation against those instances previously encountered allows the observer to assess the most likely courses of action and anticipate effectively in a timely manner.

Practice history data from expert performers reveal that vast amounts of deliberate practice are required over numerous years to attain high levels of perceptual–cognitive–motor expertise (see Ericsson et al., 1993; Williams et al., 2012b). Consequently, researchers have started to consider whether training interventions may be developed that facilitate the more rapid acquisition of perceptual–cognitive skills. The majority of researchers have focused on training advance cue utilization using relatively closed skills such as goalkeepers saving penalty kicks in soccer (Savelsbergh et al., 2010) or players attempting to return serve in tennis (Farrow and Abernethy, 2002). These interventions seek to direct attention toward the most critical cues (as determined from process measures such as gaze behavior and verbal reports) and the effectiveness of this training is assessed by comparing performance post-intervention to an earlier pre-test. In general, these training programs have reported positive findings across sports (e.g., Scott et al., 1998; Williams et al., 2003; Murgia et al., 2014).

Although researchers have highlighted the potential benefit of training perceptual–cognitive skills, at least in micro-situations (i.e., one vs. one), there have been relatively few attempts to train perception of patterns in macro-situations (i.e., full-sided games). The later observation is surprising given the fairly substantive literature base focusing on identifying the processes and mechanisms underpinning this skill (Smeeton et al., 2004; North et al., 2009, 2011). Moreover, the ability to recall and recognize patterns is considered one of the key attributes of expert performers (Abernethy et al., 2005).

In one rare exception, Gorman and Farrow (2009) attempted to train the perception of patterns in basketball. However, these authors failed to report any advantage for their experimental groups over a control group and there was no positive transfer to on-court performance. A potential limitation to their approach was the mode of presentation since the viewing perspective employed during the intervention was different to that experienced in the on-court transfer task, potentially explaining the lack of on-court improvement. Another potential limitation was the use of highly skilled participants only. The majority of researchers who have reported the benefits of perceptual–cognitive training programs have used novice or intermediate performers. The benefits of such training programs may be restricted to, or are optimized in, more novice or intermediate populations.

A challenge when devising perceptual–cognitive training interventions is how to direct attention toward the critical cues. Typically, explicit verbal instructions have been used to focus the learner’s attention on the desired display features. A series of “if-then” statements are employed to highlight how these cues related to the eventual event outcome (e.g., see Smeeton et al., 2005). Although these explicit instructional methods have produced positive training effects, it has been argued that the use of such methods can be detrimental in the long term and especially when performing under anxiety (Abernethy et al., 2012). Learning under explicit instruction is thought to result in the development of declarative knowledge, making performers prone to reinvest in this consciously controlled information when under pressure. In contrast, implicit modes of instruction seek to facilitate learning without accruing declarative knowledge, with published reports suggesting that performance is more robust when subsequently performing under pressure given the relative absence of declarative knowledge in which to reinvest (for a review, see Masters, 1992; Jackson and Farrow, 2005; Masters and Maxwell, 2008; Hill et al., 2010). In this paper, our focus was to compare different modes of implicit instruction that guided attention to relevant cues without explicitly stating how these were to be used.

It appears that methods which guide the learner’s attention, as opposed to being told explicitly, and permit performers to self-discover and learn independently are the most effective strategies to train perceptual–cognitive skills as they show both short- and long-term advantages. However, there are various means by which the attention of learners can be directed. Although Smeeton et al. (2005) used simple verbal instructions to guide attention, technology allows video footage to be edited so that additional information can be overlaid on top of the footage to direct attention to the pertinent cues. Hagemann et al. (2006) and Abernethy et al. (2012) have used transparent colored masks to highlight critical cues when anticipating shots in badminton and handball, respectively. Moreover, prompts such as arrows may be overlaid on the screen to direct attention (see Ryu et al., 2013). However, the empirical evidence supporting the effectiveness of such methods is equivocal and there is no consensus as to whether one of these strategies is better than the others or if they offer any advantages at all over simply directing attention using verbal instructions (as per Smeeton et al., 2005).

The most effective method of conveying information remains unclear and conflicting results mean there is a need to further investigate the value of perceptual-training programs. In addition, there remains a paucity of research examining whether pattern recognition skill can be trained. Also, while experts may be differentiated from less-expert counterparts on their ability to recognize patterns, it has been argued such a task is only an indirect measure of expertise and not a skill that is explicitly employed in performance contexts. A debate exists as to whether recognition simply represents a by-product of exposure to the domain and does not directly contribute to the expertise they demonstrate in the performance environment (see Ericsson and Lehmann, 1996; North et al., 2009, 2011). In light of this debate, we have participants complete an anticipation test before and after the pattern recognition training intervention to assess if any benefits of training pattern recognition transferred to what may be considered a more representative measure of expertise (cf., Mann et al., 2007).

In sum, we investigate whether it is possible to train the perceptual–cognitive skill of pattern recognition between display features (i.e., players) using soccer as the vehicle. In light of the absence of any significant effects in the study by Gorman and Farrow (2009), which used elite basketball players, we examined whether this skill was amenable to training using a more novice population group. Also, we compared the relative effectiveness of four different instructional methods. Participants were assigned to either a verbal cueing, visual cueing, video only with no cueing, or a control condition. Finally, given recent findings which have suggested recognition skill may not be as closely related to anticipation as previously thought (North et al., 2009, 2011), we examined whether the benefits of training pattern recognition transfers to improvements in anticipation accuracy. Since previously researchers have shown a variety of instructional approaches to be effective in training perceptual–cognitive skill in micro-contexts, we hypothesized that all three experimental conditions would improve recognition performance from pre- to post-test in comparison to a control group. Also, we expected the verbal cueing and visual cueing groups to improve more than the video only group given that their attention was being directed to those features identified as most important in successful recognition judgments (see North et al., 2009, 2011; Williams et al., 2012a). Since only a few researchers have directly tested different instructional methods, producing contradictory results, we had no *a priori* hypothesis as to whether visual or verbal cueing would be more effective in training recognition. As knowledge and awareness of patterns of play has consistently been identified as a characteristic of expert performers (Abernethy et al., 2005; Williams et al., 2006), and even published reports suggesting the skill may not be central to anticipation performance (North et al., 2009, 2011) report positive correlations between the two, our final hypothesis was that successfully training the ability to recognize patterns of play would result in improvements in anticipation accuracy.

## Materials and Methods

### Participants

Altogether, 64 amateur soccer players volunteered to participate. The performance of participants was rank-ordered based on their pre-test recognition accuracy scores, following which participants were then assigned to one of four equally matched groups of *N* = 16: control (*M* age = 19.5 years, *SD* = 2.07); visual attention guided (*M* age = 20.2 years, *SD* = 3.24); verbal instruction guided (*M* age = 19.8 years, *SD* = 3.67); and video only (*M* age = 20.9 years, *SD* = 2.47). Participants were considered as amateurs if they had only played soccer at recreational or school level. Participants reported having played soccer at this level for an average of 9.24 years (*SD* = 2.55). All reported normal or corrected to normal visual function and none reported color blindness. The research was conducted according to the ethical guidelines and approval of the second author’s institution. Participants provided written informed consent and were free to withdraw at any stage.

### Test Films

We used three different test films. An anticipation test film, a perceptual training test film, and a recognition test film. All test films used video footage which was recorded using a fixed, tripod-mounted video camera (Canon XM-2, Tokyo, Japan) in a raised position (approximate height 9 m) set back behind the goal (approximate distance 15 m). The camera position ensured that all players were visible at all times and that information was not excluded from wide areas. Although the raised viewing perspective is different to that which players would typically experience during game situations, construct-validity has previously been established for the approach. When using the same viewing perspective, expert–novice differences have been reported using recognition (North et al., 2011), recall (Abernethy et al., 2005), situational probability (Ward and Williams, 2003), and anticipation (North et al., 2016) paradigms. All test films comprised of a number of separate clips, each showing a developing pattern of play which culminated in a penetrative attacking pass to a teammate. All the action sequences used showed patterns of play developing in the direction of the camera (i.e., coming toward the participant) and were all “structured” in nature. Clips were classified as being structured on the basis of three expert coaches independently rating a battery of clips as being either low or high in structure using a Likert-type scale (0 = very low in structure, 10 = very high in structure). The clips rated most highly for structure were those judged to be most representative of tactics, strategies, and plans that would typically be observed in attacking play. Only clips with a mean rating above 7 were used.

#### Recognition Test Films

The action sequences used for the recognition test films were sampled from three English Premier League reserve team matches. The recognition test was comprised of a viewing phase and a recognition phase. Each individual clip in both viewing and recognition phases was 7 s in duration. The initial 2 s showed a static image of the first frame in the sequence, during which participants were cued to the location of the ball by a red circle. The clip then played normally for 5 s, showing a developing pattern of play before it occluded to black. There was then a 3 s inter-trial interval after which the next 7 s clip played in the same fashion. Both viewing and recognition test films contained 40 clips, however, for the recognition test film 20 of these were also present in the initial viewing test film and 20 were novel.

#### Anticipation Test Film

**FIGURE 1:**
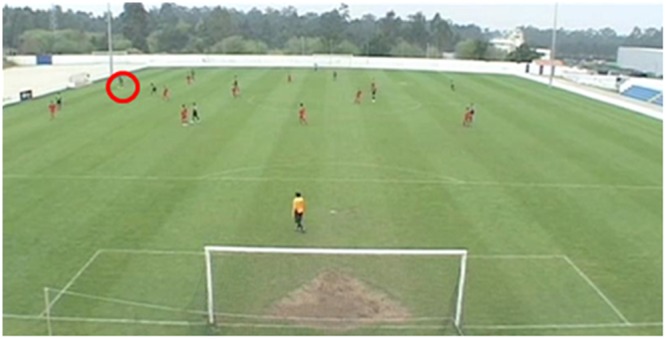
An example of a freeze-frame shown prior to the onset of a clip with the starting position of the ball indicated by a red circle.

**FIGURE 2:**
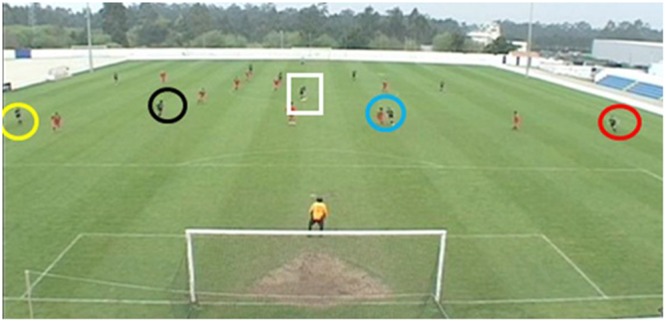
An example of a freeze-frame shown at the end of each clip in the anticipation paradigm with the passing options highlighted by yellow, black, blue, and red circles. Note: White square shown here is for illustrative purposes to highlight ball location and was not used in the actual test film.

At the start of each clip in the anticipation phase, participants were shown a freeze-frame of the clip’s opening frame for 2 s. During this time, a red circle was shown on the screen to cue participants as to the ball’s location. The red circle then disappeared and the clip played normally, showing 6 s of action in which possession started in the defensive half (that furthest away from the participant) and ended in the attacking half (that nearest the participant). Each clip stopped when the player in possession was about to make a penetrative pass to a teammate in an attacking position. The final frame when the clip ended was paused and presented to participants for 2-s, during which time possible passing options were highlighted using red, blue, black, and yellow squares. The clip then occluded to a black screen and the next clip commenced after a 5 s inter-trial interval. In total, there were 24 clips in the anticipation test film with each being presented for a total of 10 s. An example of the first and final frames of a clip used in the anticipation test film is presented in **Figures 1, 2**.

#### Perceptual Training Film

The match footage used in the perceptual training films was taken from a sample of two Football Association under 18 years Youth Cup matches. In total, there were 120 clips spread over four perceptual training sessions. Participants were assigned to one of four different perceptual training groups. The precise nature of footage in the perceptual training film was dependent on which group participants were assigned to following the pre-test.

### Apparatus

Film clips were presented using a DVD player (Panasonic, DMR-E50, Osaka, Japan) and projector (Sharp, XG-NV2E, Manchester, United Kingdom) to project images onto a 9′ × 12′ screen (Cinefold, Spiceland, IN, United States) at a rate of 25 frames/s with XGA resolution. Verbal instructions were recorded onto a dictaphone and transferred onto test films using video editing software (Adobe Premiere, Adobe Systems Incorporated, San Jose, CA, United States). The same video editing software was used to create the test films and insert freeze-frames in sequences. To highlight the ball and players of interest, the Microsoft Paint Program (Microsoft Corporation 2010, Redmond, WA, United States) was used.

### Procedure and Tasks

Participants completed pre- and post-tests to assess anticipation and recognition performance which were separated by 2 weeks. During the intervening 2 weeks, participants completed the perceptual training program spread over four separate sessions, with approximately 2.5 days between each perceptual training session.

#### Pre-tests

Participants initially completed the recognition test. This involved participants being presented with the viewing test film which comprised of 40 individual clips. Participants were informed that the ball’s starting location would be highlighted by a red circle, after which the clip would play normally and show a developing attacking sequence that culminated in a player being about to make a forward attacking pass, but that the clip would occlude before this pass was played. Participants were instructed to watch the clip as if they were playing in the match as a central defensive player, but that no specific response was required. After the viewing film had been presented there was a 10 min comfort break. Participants were then presented with the recognition film which comprised of 40 clips. The participants were told that some of the clips in the recognition film had been included in the viewing phase and that others were novel; their task was to make a recognition decision for each clip as to whether they had seen it in the viewing film or not. Participants were instructed to watch each clip for its full duration before making a recognition response (yes or no) by writing down their answer using pen and paper. When each clip was occluded in the recognition phase, participants were presented with a “Respond Now” image on the screen and were instructed to respond quickly and accurately. The recognition test took approximately 20 min to complete.

After completing the recognition test, participants were provided with another 10 min break during which they completed a short questionnaire that requested demographic information as well as information about their practice history and involvement in soccer. Participants then completed an anticipation test. Participants were informed they would be presented with a further 24 clips showing developing patterns of play, which culminated in a player about to make an attacking pass and that they should watch the clips as if they were a central defensive player. The participants were told that a red circle would highlight the ball’s position at the start of the clip before playing and then pausing on the final frame of the sequence. Participants were told that four different passing options would be highlighted using colored circles and that their task was to select the player they thought was most likely to receive the ball by writing down the respective colored circle on a pen and paper response sheet. The final frame was paused for 2 s, after which the message “Respond Now” was presented on the screen. Participants were instructed to respond quickly and accurately. The anticipation test took approximately 10 min to complete.

#### Perceptual Training

Participants were allocated to one of four equally matched perceptual-training groups based on their pre-test recognition scores. There were four training sessions, each comprising of 30 clips with an inter-trial interval of 5 s, with each training session taking approximately 20 min to complete. Participants were not required to make any responses during the training sessions, but they were informed to watch the clips and pay attention to any instructions or guidance provided within these session.

##### Verbal instruction group

When the first frame was presented and “frozen” for 2 s to cue participants to the location of the ball, participants in this group were provided with verbal instructions about where they should direct their attention during the clip. The instruction provided was based on findings reported by Williams et al. (2006) and North et al. (2009), with participants being told to focus their attention on the positions and movements of central attacking players without explicitly stating the purpose of the movements that were to be made or exactly what information this might convey. Generic verbal instructions were provided at the onset of the sequence to focus on these specific players with subsequent verbal instructions individually tailored for each clip. For example, a clip in which two strikers would move in order to create space for another teammate would play as normal after the initial 2 s freeze-frame, before a second freeze-frame would be inserted at an appropriate point in order for verbal instructions to be provided to highlight the specific movements and runs of interest. Once the verbal instructions had been provided the clip resumed and played as normal. Each clip contained 6 s of dynamic activity although the total presentation time varied due to each clip being individually tailored with freeze-frames and additional verbal instructions inserted as appropriate.

##### Visual guidance group

**FIGURE 3:**
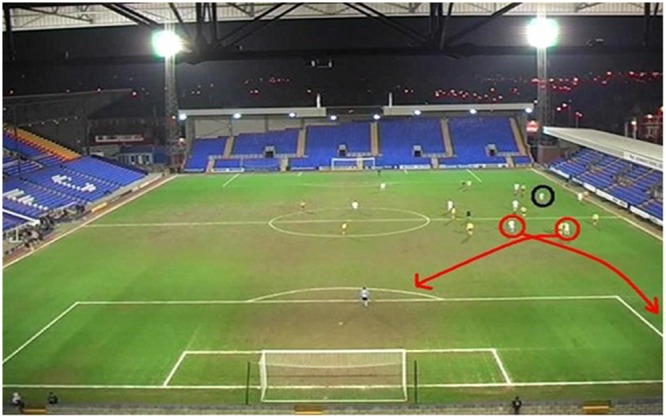
An example of a freeze-frame from the visual attention intervention which highlights ball location and the position and subsequent movement of the two central attacking players.

The clips used, and their order of presentation, were the same as in the verbal instruction training group. During the initial 2 s freeze-frame in which ball location was identified using a black circle, participants in this group were cued as to the most important players and where they should direct their attention using red circles (to highlight the players) and red arrows (to highlight their movements). The visual cues to guide attention were all presented during the freeze-frame only so as to avoid potentially obstructing information once clips played normally. To ensure consistency, the same players were highlighted as in the verbal instruction group and each clip was played for the same length of time with the same number of freeze-frames inserted for the same length of time. An example of a freeze-frame from the visual guidance group is shown in **Figure 3**.

##### Video only group

In this group, participants were presented with the same clips, in the same order, as in the verbal instruction and video guidance groups. As with these groups, participants were initially presented with a 2 s freeze-frame during which the position of the ball was highlighted using a red circle. Participants in this group though received no further information and after the initial 2 s freeze-frame the clip played normally for 6 s before the screen was occluded.

##### Control group

Participants in this group were not exposed to any training and only completed pre- and post-recognition and anticipation tests.

#### Post-tests

Participants completed recognition and anticipation post-tests 2 days after the final perceptual training session. The post-test recognition and anticipation tests were conducted following the same procedures used for the pre-tests. To prevent familiarity bias and expectancy effects, the order of presentation for clips was changed from the pre-test and the clips that were repeated in the recognition test film were different to those in the pre-test. Also, the order of clips was changed for the anticipation paradigm.

### Dependent Measures and Data Analysis

The data were analyzed based on signal detection theory. This analysis method is used to measure the effectiveness of participants in distinguishing meaningful signals that may be present in displays from non-meaningful noise. Signal detection theory provides two dependent measures which were used to analyze recognition performance; a parametric measure of sensitivity (*d*′) and criterion (*c*) which is a measure of response bias (Green and Swets, 1966). The measure of sensitivity (*d*′) assesses discriminability: how well two conditions can be distinguished from one another (signal present or absent). The larger the *d*′ value the more sensitive a person is in discriminating between signal present and signal absent stimuli, while a value of 0 indicates chance (i.e., guessing) performance. Criterion (*c*) measures bias and refers to the extent to which one response (i.e., responding yes or no) is more probable than the other. If the *c* value is negative it indicates a bias toward “yes” responses (resulting in more “hits,” but also more “false alarms”), whereas if *c* is a positive value then it indicates the participants favor a bias to “no” responses, with fewer hits and fewer false alarms (MacMillan, 2002).

Anticipation performance was measured by dividing the total number of correct judgments by the total number of trials (*n* = 24) and then multiplying by 100 to create a percentage accuracy score. The data for *d*′, *c*, and anticipation accuracy were analyzed using separate two-way mixed-design ANOVAs in which the between participant factor was Group (verbal instruction vs. visual guidance vs. video only vs. control) and the within participants factor was Time of Test (pre-test vs. post-test).

Prior to conducting the analyses, all data were tested for normality using the Shapiro–Wilks test. Partial eta squared ($\eta_{p}^{2}$) values are provided as a measure of effect size for all main effects and interactions and, where appropriate, Cohen’s *d* measures are reported for comparisons between two means. For repeated measures, violations of sphericity were corrected by adjusting the degrees of freedom using the Greenhouse–Geisser correction when the sphericity estimate was less than 0.75 and the Huynh–Feldt correction when greater than 0.75 (Girden, 1992). The alpha level for significance was set at *p* < 0.05.

## Results

### Recognition Performance

**FIGURE 4:**
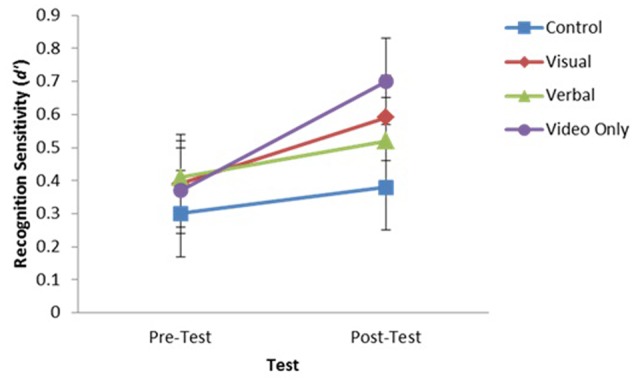
Mean recognition sensitivity (*d*′) with error bars for each experimental group at pre and post recognition tests.

An analysis of *d*′ revealed a significant main effect of Time of Test on recognition sensitivity, *F*(1,60) = 16.53, *p* < 0.05, $\eta_{p}^{2}$ = 0.216. Participants were more sensitive in their recognition decisions at post-test (*M* = 0.55, *SD* = 0.5) than pre-test (*M* = 0.37, *SD* = 0.52), *d* = 0.34. However, the effect of Group, *F*(3,60) = 0.49, *p* > 0.05, $\eta_{p}^{2}$ = 0.02, and the Group × Time of Test interaction, *F*(3,60) = 1.53, *p* > 0.05, $\eta_{p}^{2}$ = 0.07, was not significant. The mean recognition sensitivity scores for each experimental group at pre- and post-tests are shown in **Figure 4**.

For *c*, ANOVA showed that the effects of Time of Test, *F*(1,60) = 0.39, *p* > 0.05, $\eta_{p}^{2}$ = 0.01, Group, *F*(3,60) = 0.31, *p* > 0.05, $\eta_{p}^{2}$ = 0.02, and the Group × Time of Test interaction, *F*(3,60) = 1.41, *p* > 0.05, $\eta_{p}^{2}$ = 0.07, were all non-significant. These data demonstrate that neither the experimental group or time of test made participants more biased to responding : “yes” or “no” when making recognition decisions.

### Anticipation Accuracy

The ANOVA revealed no main effect of Time of Test, *F*(1,60) = 3.61, *p* > 0.05, $\eta_{p}^{2}$ = 0.06, or Group, *F*(3,60) = 0.22, *p* > 0.05, $\eta_{p}^{2}$ = 0.01. The Group × Time of Test interaction was non-significant, *F*(3,60), = 0.41, *p* > 0.05, $\eta_{p}^{2}$ = 0.02.

## Discussion

In this paper, we had three aims. First, we examined whether participants were able to improve the perceptual–cognitive skill of recognizing patterns between features following a perceptual–cognitive training intervention. Also, we tested the relative effectiveness of different instructional approaches to train this skill. Finally, given debate as to the importance of recognition in anticipation, we examined whether our training interventions, which focused on enhancing pattern recognition would transfer to improvements in anticipation accuracy.

We hypothesized that recognition performance would improve from pre- to post-test (cf., Farrow and Abernethy, 2002; Williams et al., 2003; Smeeton et al., 2005). This hypothesis was supported. Participants became more sensitive in distinguishing previously seen from novel patterns on the post- relative to the pre-test. However, contrary to our second hypothesis, there was no main effect of group and no Group × Time of Test interaction, indicating that the mode of instruction did not affect how well participants learned to recognize patterns. This pattern of findings closely mirrors that reported by Gorman and Farrow (2009) who showed significant improvements from pre- to post-test but reported that the improvements observed in the training groups did not differ when compared with the placebo and control groups. It is often suggested that the absence of significant effects is due to the small sample size and relatively short intervention period. However, in the current study, we present one of the most extensive perceptual training interventions conducted in the literature. The intervention period (2 weeks) was longer than, or comparable to, other studies which have trained perceptual skills and reported significant effects (e.g., 3 days, Abernethy et al., 2012; 7 days, Hagemann et al., 2006; 7 days, Ryu et al., 2013; 3 weeks, Serpell et al., 2011; and 45 min, Williams et al., 2002). Over the course of the intervention period, participants were exposed to 120 training trials which is a higher number than employed in other similar perceptual training studies (e.g., 64 trials, Ryu et al., 2013; 40 trials, Serpell et al., 2011; 8 trials; Williams et al., 2002; and 30 trials, Smeeton et al., 2005).

Our intervention failed to replicate the benefits evident using perceptual–cognitive training programs designed to improve the ability to use advance postural cues. However, perceiving patterns between independent display features (i.e., players) and perceiving advance postural cues (and potentially relations between these interrelated features) are two distinct perceptual–cognitive skills. Perceiving global patterns between features may represent a higher order and more strategic skill, whereas perceiving postural cues could represent a lower-order process. As a consequence, the higher-order, more strategic skills may require an extended training program with more prolonged exposure to stimuli and game patterns in order to see the same extent of performance improvements that are observed when training the ability to perceive more localized cues. In both Gorman and Farrow’s (2009) study and the current paper, there are trends in the data (albeit non-significant) for training intervention groups to improve in spite of relatively short-term interventions. Although the duration of our intervention was comparable (or longer) to that reported in other published reports where attempts have been made to train the pick-up of advance postural cues, researchers should seek to undertake more longitudinal interventions to investigate whether the trends observed lead to significant differences over time.

The information we highlighted using our training interventions was driven by research which had identified the central attacking players, and specifically the relative motion between these features, as being the critical information to convey structure and meaning in order to perceive patterns in dynamic, interactive displays (see Williams et al., 2006, 2012a; North and Williams, 2008; North et al., 2009, 2011). However, in seeking to ensure participants were attending to these critical features we provided a lot of detail (either through verbal instruction or visual highlighting) that was tailored to each individual sequence. It is possible we were overly prescriptive with the information provided and unintentionally we may have promoted an explicit style of learning. More pronounced benefits of the intervention may have been seen had participants only had their attention oriented to these features at the outset and then subsequently been allowed to discover the movement patterns and relations for themselves. Such an intervention would have likely promoted a more implicit style of learning, which is considered preferable and more advantageous than learning explicitly (Magill, 1998; Smeeton et al., 2005). Orienting attention toward the most critical features at the outset before subsequently allowing the sequence to play would have the advantage of allowing relative motion information to emerge more clearly. The level of detail we sought to present necessitated that “freeze-frames” be inserted in video sequences, which in itself is likely to have disturbed or distorted the relative motion information emerging. Williams et al. (2012a) have demonstrated that although the relationships between display features are important, specifically it is the relative motion information emerging through dynamic interactions between these features that are critical. The use of “freeze-frames” to highlight features may have served to prevent participants from extracting this critical source of information, in turn impairing their ability to perceive patterns within the displays. An intervention which simply directed attention before allowing sequences to play uninterrupted would both encourage a more implicit style of learning and enhance the potential for the critical relative motion information to emerge.

The highly prescriptive approach used to orientate attention may have resulted in participants adopting a narrow focus of attention (cf., Nideffer, 1976). While a narrow focus of attention can be advantageous in situations where the visual information is largely invariant (Nougier et al., 1991), in dynamic contexts, such as soccer, where visual information is highly variable in nature, a broader focus of attention is considered preferable (Ripoll, 1988). Although the relative motions between central attacking players have been demonstrated as critical information sources, it is likely that participants need an awareness of how these more localized relations fit within the broader and more global pattern.

Our final aim was to examine whether any improvements in pattern recognition would transfer to improvements in anticipation. We did not observe any change in anticipation accuracy from pre- to post-test. Gorman and Farrow (2009) similarly did not report any main effects or interactions. It may be that perception of patterns does not contribute to anticipation, but rather that it is merely a by-product of task experience (cf., Ericsson and Lehmann, 1996), or at the very least its contribution to anticipation is less than has been previously argued. A number of distinct perceptual–cognitive skills contribute to anticipation and decision-making (see Vaeyens et al., 2007; Williams and Ward, 2007; Roca et al., 2013). The relative importance of these perceptual–cognitive skills varies as a function of the task constraints under which one is performing. In soccer, Roca et al. (2013) and North et al. (2016) have demonstrated that when the ball is far away from the performer, they seek to perceive patterns between features to inform their decision-making. In contrast, as the ball moves closer to the performer, attention switches to utilizing postural cues, with perception of patterns between players becoming less important. The stimuli used in the anticipation paradigm in the present study all showed action sequences where the final pass was about to be made in relatively close proximity to the participant. Therefore, the task constraints used in this study may have dictated that participants seek to process postural cues rather than perceive patterns. The nature of the clips (i.e., a raised viewing perspective) is likely to have made it difficult to extract fine postural cues, meaning that while participants may have looked to use this source of information, their ability to do so will have been impaired and so this may explain why anticipation accuracy did not improve. Alternatively, the skill of perceiving patterns between features may not have been required to inform anticipation judgments in this study and may be one explanation for the lack of transfer to anticipation accuracy following the perceptual training intervention. It may also be that the methods employed to examine transfer lack sufficient sensitivity to capture any benefits that may emerge. In future, researchers may wish to consider supplementing the anticipation paradigm used here with some more direct field-based measures of performance using match analysis data or the ratings of expert coaches on *in situ* assessment of anticipation and decision-making using behavioral assessment scales (e.g., see French and Thomas, 1987; Oslin et al., 1998).

When assessing transfer effects, an important factor to consider is viewing perspective. While the raised viewing perspective used in this study has distinguished skilled and less-skilled performers in recall (Abernethy et al., 2005), recognition (Williams et al., 2006), situational probability (Ward and Williams, 2003), and anticipation tasks (North et al., 2016) it nevertheless provides a very different perspective to that which players would encounter on the field. The encoding specificity principle (Tulving and Thompson, 1973) gives reason to be skeptical that any training benefits using such third person perspectives might transfer to field environments given the clear differences in perceptual information during encoding and retrieval processes across these two contexts. A potentially fruitful area for researcher and practitioners is the use of immersive technologies (such as virtual and augmented reality), which can more faithfully represent the perceptual variables experienced in performance environments.

A final potential issue is that all of the conditions employed a perception-only mode of response (i.e., pen and paper). Some researchers argue for the need to ensure perception and action are tightly coupled when studying perceptual–cognitive–motor skills (see Mann et al., 2010) to ensure both ventral and dorsal streams are engaged and that tasks more faithfully represent those undertaken in performance environments. However, there are numerous previous examples whereby perceptual–cognitive skills have been improved using uncoupled training methods (e.g., see Farrow and Abernethy, 2002; Williams et al., 2002) and there is evidence that the motor system remains engaged during perception-only tests (e.g., see Urgesi et al., 2006). Furthermore, in applied contexts, some elite sporting organizations are opposed to athletes engaging in overt physical practice beyond formally scheduled training and competition situations due to increasing concern over overuse injuries through excessive physical exertion (see Pimenta et al., 2017; van Mechelen et al., 2017). Also, perception-only interventions have practical utility since they can be employed when players are injured or in transit to and from training and matches.

## Conclusion

Wu attempted to train the perceptual–cognitive skill of recognizing patterns between display features. Although our analyses revealed non-significant effects, we have employed novel and innovative methods and presented a foundation for follow-up research. We have raised a number of important points that should be valuable and informative for scientists and practitioners when designing interventions to improve perception of patterns in future. A body of research now exists which identifies critical information sources for pattern perception (see North and Williams, 2008; North et al., 2009, 2011; Williams et al., 2012a). When seeking to enhance pattern perception by improving awareness of these critical information sources, we suggest that in future, researchers need to employ more longitudinal interventions. Finally, any interventions should not disturb relative motion information and any attempts to examine transfer should ensure the task constraints encourage the perception of patterns between features over and above any other perceptual–cognitive skills that performers may have available.

## Ethics Statement

This study was carried out in accordance with the recommendations of Liverpool John Moores University ethics committee (ethics approval number: 09/SPS/010) with written informed consent from all participants. All participants gave written informed consent in accordance with the Declaration of Helsinki. The protocol was approved by the Liverpool John Moores University ethics committee (ethics approval number: 09/SPS/010).

## Author Contributions

JN, EH, and AW study design and development; EH data collection; JN, EH, and AW data analysis and interpretation; and JN, EH, and AW writing of manuscript.

## Conflict of Interest Statement

The authors declare that the research was conducted in the absence of any commercial or financial relationships that could be construed as a potential conflict of interest.
